# Cardiac troponin sensing *via* carbon dot-based optical biosensors: advances and clinical opportunities

**DOI:** 10.1039/d6ra05727f

**Published:** 2026-10-07

**Authors:** Shilan A. Fatah, Aryan A. Fatah, Elyan A. Abdulla, Sanaa K. Ali, Rzgar Sirwan Raza, Sameera Sh. Mohammed Ameen, Khalid M. Omer

**Affiliations:** a Department of Pharmacy, Kurdistan Technical Institute Sulaimani KGR 46001 Iraq shilan.fatah@kti.edu.iq; b Medical Laboratory Department, Nawa Hospital Sulaimaniyah 46001 Kurdistan Region Iraq; c Department of Chemistry, College of Science, University of Sulaimani Sulaymaniyah Kurdistan Region 46002 Iraq khalid.omer@univsul.edu.iq; d Department of Artificial Intelligence and Data Science, College of Science and Technology, University of Human Development Sulaymaniyah Kurdistan Region of Iraq Iraq; e Department of Chemistry, College of Science, University of Zakho 42002 Zakho Kurdistan region Iraq

## Abstract

Cardiovascular diseases, particularly acute myocardial infarction (AMI), remain a leading cause of mortality worldwide, necessitating rapid and accurate diagnostic strategies. Cardiac troponins, especially cardiac troponin I (cTnI) and T (cTnT), are established as gold-standard biomarkers for myocardial injury; however, conventional detection methods suffer from limitations in sensitivity, time consumption, and requirement of complex instrumentation. In recent years, carbon dots (CDs)-based optical biosensors have emerged as promising alternatives due to their excellent photoluminescence properties, biocompatibility, and tunable surface chemistry. This review systematically discusses the role of CDs in fluorescence, colorimetric, and chemiluminescence-based detection of cardiac troponin, highlighting their sensing mechanisms, analytical performance, and integration into portable microfluidic and point-of-care platforms. Furthermore, recent advancements, including Förster resonance energy transfer (FRET)-based systems, nanozyme-assisted assays, and multiplex chemiluminescence platforms, are critically evaluated. Finally, current challenges, limitations, and future perspectives are addressed to provide insights into the development of next-generation CDs-based diagnostic technologies for early and reliable cardiovascular disease detection.

## Abstract 2

Cardiovascular diseases, particularly acute myocardial infarction (AMI), remain a leading cause of mortality worldwide, necessitating rapid and accurate diagnostic strategies.

## Graphical abstract

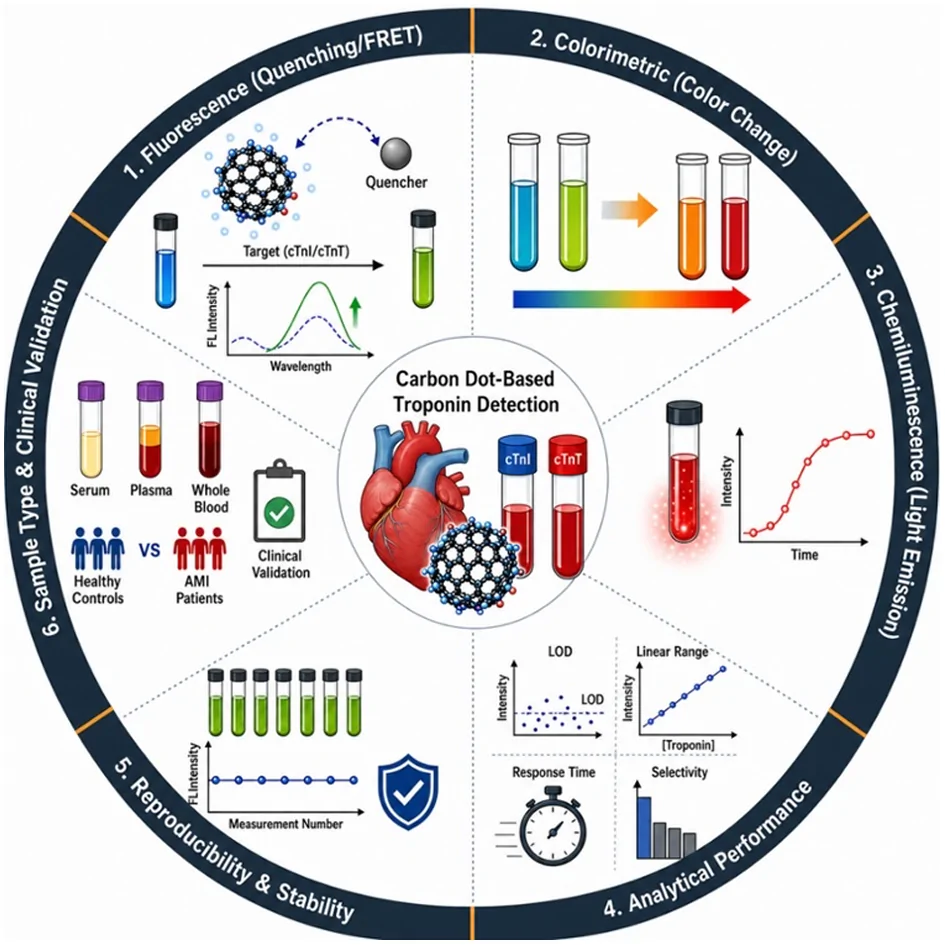

## 1. Introduction

Cardiovascular diseases (CVDs) remain the leading cause of mortality worldwide, with acute myocardial infarction (AMI), representing one of the most critical and life-threatening conditions.^1^ Early and accurate diagnosis of AMI is essential, as timely clinical intervention significantly improves patient survival and reduces long-term complications.^2^ Among the available cardiac biomarkers, cardiac troponins, particularly cTnI and cTnT, are widely recognized as the gold standard due to their high specificity and sensitivity for myocardial injury.^3^ Following cardiac damage, troponin levels increase within 3–6 hours and remain elevated for several days, providing a clinically reliable diagnostic window.^4^ Despite their clinical importance, conventional detection techniques, including enzyme-linked immunosorbent assays (ELISA), electrochemiluminescence assays, and fluorescence immunoassays, are limited by relatively long assay times and dependence on sophisticated instrumentation.^5^ These limitations restrict their applicability in rapid, point-of-care diagnostics, particularly in emergency and resource-limited settings.^6^ To overcome these challenges, nanomaterial-based biosensors offer a promising solution for the rapid and sensitive detection of cardiac biomarkers. Materials such as metallic nanoparticles (gold, silver, platinum),^7^ semiconductor nanomaterials (quantum dots, metal oxides),^8^ magnetic nanoparticles, metal–organic framework (MOFs),^9,10^ and advanced 2D materials (MXenes)^11,12^ are widely used because of their large surface area, high conductivity, catalytic properties, and tunable optical features.^13^ These characteristics improve signal transmission, biomolecule attachment, and sensor sensitivity and selectivity, allowing detection of biomarkers at very low concentrations. As a result, nanomaterial-based sensors are increasingly important for point-of-care testing and early diagnosis of cardiovascular diseases.^14^ Among various nanomaterials, CDs have gained considerable attention due to their unique physicochemical and optical properties.^15^ CDs exhibit strong and tunable photoluminescence, high photostability, excellent biocompatibility, and versatile surface functionalization.^16^ These features enable CDs to serve as efficient signal transducers in optical biosensing platforms.^17^ Recent advances have demonstrated that CDs-based biosensors can achieve high sensitivity and rapid detection of cardiac troponins through multiple optical strategies.^18^ Fluorescence-based systems utilizing FRET, colorimetric assays based on nanoparticle aggregation, and chemiluminescence platforms with signal amplification have shown significant improvements in analytical performance.^19^ Moreover, integration with microfluidic devices and portable platforms has enabled the development of user-friendly point-of-care diagnostic systems.^20^

Nevertheless, despite these significant advances, several critical challenges continue to hinder the practical and clinical translation of CDs-based biosensors.^21–23^ One major limitation is the absence of standardized and reproducible synthesis protocols for CDs, which often results in variations in particle size, surface chemistry, optical properties, and sensing performance. Moreover, although many reported biosensing systems demonstrate excellent analytical sensitivity under controlled laboratory conditions, their reliability and stability in complex biological matrices such as serum and whole blood remain insufficiently investigated. Another important limitation is that most currently available platforms are designed for single-analyte detection, whereas modern clinical diagnostics increasingly require multiplexed biomarker analysis to improve diagnostic accuracy and reduce false-positive or false-negative outcomes.

Several reviews have addressed cardiac troponin sensing, nanomaterial-based biosensors, or carbon-dot-based biosensing from broader perspectives; however, their scopes differ substantially from the focus of the present review. For example, Du *et al.* provided a comprehensive overview of troponin analysis for the early diagnosis of cardiovascular diseases, covering diverse analytical strategies and emphasizing the progress, opportunities, and challenges associated with troponin detection.^7^ Fathil *et al.* reviewed cardiac troponin sensing based on semiconducting nanomaterials, with particular emphasis on charge-transduction approaches and semiconductor-based devices.^8^ More recently, carbon-dot-based reviews have systematically discussed the use of CDs in biosensing and disease-biomarker detection, including fluorescence, colorimetric, electrochemical, and electrochemiluminescence strategies, but without specifically focusing on the optical detection of cardiac troponins.^24^ In contrast, the present review specifically focuses on CDs-based optical biosensors for cardiac troponin detection, with particular emphasis on fluorescence, För FRET, inner-filter effect (IFE), colorimetric, chemiluminescence, and portable sensing strategies. Beyond summarizing analytical performance, this review critically compares sensing mechanisms, recognition elements, signal-transduction pathways, detection characteristics, limitations, and potential clinical applicability. Particular attention is also given to the progression from conventional laboratory-based assays toward portable, microfluidic, multiplexed, and point-of-care platforms. Thus, this review provides an integrated perspective connecting the properties and optical functions of CDs with cardiac troponin detection, while identifying the key technological gaps that must be addressed for reliable clinical translation. The literature search was conducted using Scopus, Web of Science, PubMed, and Google Scholar. The review primarily covers studies published from 2011 to August 2026. Earlier relevant publications were also included where necessary to provide fundamental background and historical context.

To further clarify the novelty of the present review, Table 1 compares its scope with representative previous reviews addressing cardiac troponin sensing, semiconductor nanomaterials, and carbon-dot-based biosensing. Previous studies have generally focused either on cardiac troponin detection using diverse analytical technologies, semiconducting nanomaterials, or the broader application of carbon dots in biosensing. In contrast, the present review specifically integrates the optical functions of carbon dots with cardiac troponin recognition and systematically evaluates fluorescence, FRET, IFE, colorimetric, chemiluminescence, and emerging smartphone-assisted sensing strategies. In addition to analytical performance, particular emphasis is placed on the evolution of these platforms toward portable, microfluidic, multiplexed, and point-of-care formats, as well as the remaining barriers related to reproducibility, clinical validation, standardization, and regulatory translation. Therefore, the present review provides a more focused and clinically oriented framework for understanding the development of CD-based optical biosensors for cardiac troponin detection (Fig. 1).

**Table 1:** Comparison of previous reviews with the scope and contribution of the present review^a^

Clinical translation	Critical comparison of sensing mechanisms	POC/portable integration	Optical CD-based sensing	Cardiac troponin-specific	Detection strategies	Materials covered	Main focus	Review
Discussed	Limited	Limited	✗	✓	Multiple analytical methods	Various analytical platforms	Troponin analysis for cardiovascular diagnosis	7
Discussed	✓	Limited	Partial	✓	Mainly charge-transduction approaches	Semiconductor nanomaterials	Cardiac troponin sensing using semiconducting nanomaterials	8
General	General	Partial	Partial	✗	Fluorescence, colorimetric, electrochemical, ECL	Carbon dots	Carbon dots in biosensing and disease biomarkers	24
✓	✓	✓	✓	✓	Fluorescence, FRET, IFE, colorimetric, chemiluminescence, smartphone-assisted	Carbon dots and CD-based hybrids	CD-based optical biosensors for cardiac troponin detection	Present review

a Distinct contribution of the present review | focused synthesis of CD optical transduction mechanisms specifically for cTn detection | CDs and CD-based hybrid systems | FRET, IFE, fluorescence, colorimetric, chemiluminescence and portable approaches | ✓ | ✓ | ✓ | mechanism–performance–limitation comparison | clinical translation, standardization and future POC development.

**Fig. 1:**
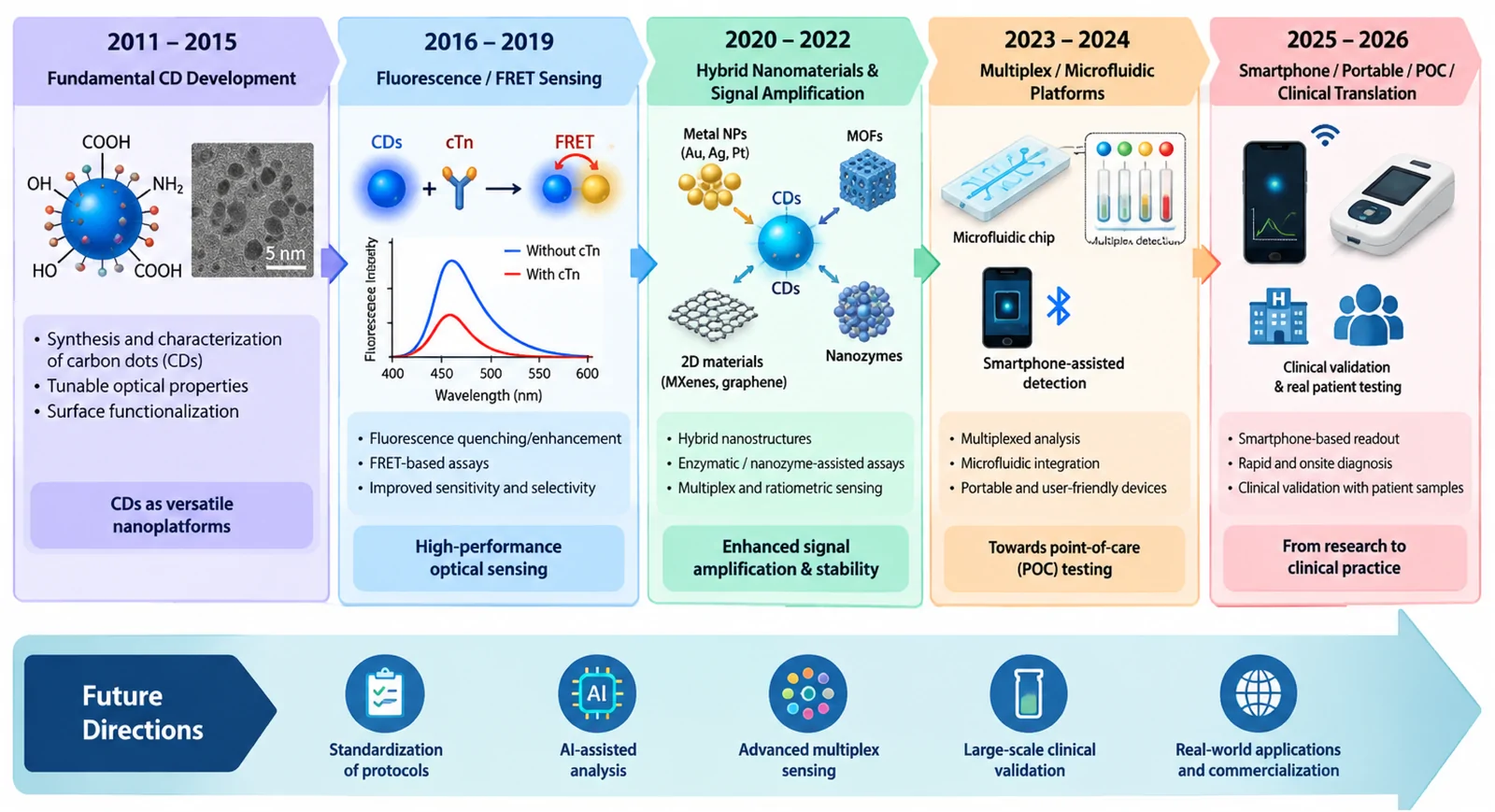
Roadmap illustrating the evolution of carbon dot-based optical biosensors for cardiac troponin detection, from fundamental carbon-dot development and fluorescence-based sensing to hybrid nanomaterials, microfluidic and multiplexed platforms, and emerging smartphone-assisted point-of-care and clinically oriented applications. Future directions include standardization, AI-assisted analysis, advanced multiplexing, large-scale clinical validation, and real-world translation.

## 2. Troponin

Cardiac troponins are essential biomarkers for diagnosing acute coronary syndromes, with levels above the 99th percentile upper reference limit indicating myocardial injury, and a dynamic rise and/or fall combined with clinical or imaging evidence confirming acute myocardial infarction.^25^ Although elevated troponin usually reflects true cardiac damage, rare cases of falsely elevated levels, known as spurious hypertroponinemia, can occur in the absence of myocardial ischemia. These false elevations may result from pre-analytical factors (such as improper sample collection or handling), analytical factors (including assay interference or antibody-related issues), or post-analytical factors (errors in reporting or interpretation).^26^ Recognizing these potential causes is important to avoid misdiagnosis and ensure appropriate clinical decision-making.^26^ cTnI is a highly specific biomarker released into the bloodstream after cardiac injury and plays a crucial role in early heart attack diagnosis. However, conventional detection methods often require large blood volumes and may have limitations in sensitivity and selectivity.^26^

Troponin is a Ca^2+^-sensitive protein complex that regulates muscle contraction by controlling the position of tropomyosin on thin filaments. It consists of three subunits: TnC (binds calcium), TnI (inhibits contraction), and TnT (anchors the complex to tropomyosin).^27^ Structural analysis revealed that TnT spans the head-to-tail junction of tropomyosin filaments, while the globular head region of troponin binds at a distant site along the tropomyosin molecule, explaining how the complex structurally stabilizes and regulates the thin filament during muscle contraction.^28^Scheme 1 shows the role of the troponin complex (TnC, TnI, and TnT) in regulating muscle contraction on the actin filament. When Ca^2+^ binds to TnC, tropomyosin moves to expose myosin-binding sites and allow contraction, while removal of Ca^2+^ blocks the sites again and causes muscle relaxation.

**Scheme 1:**
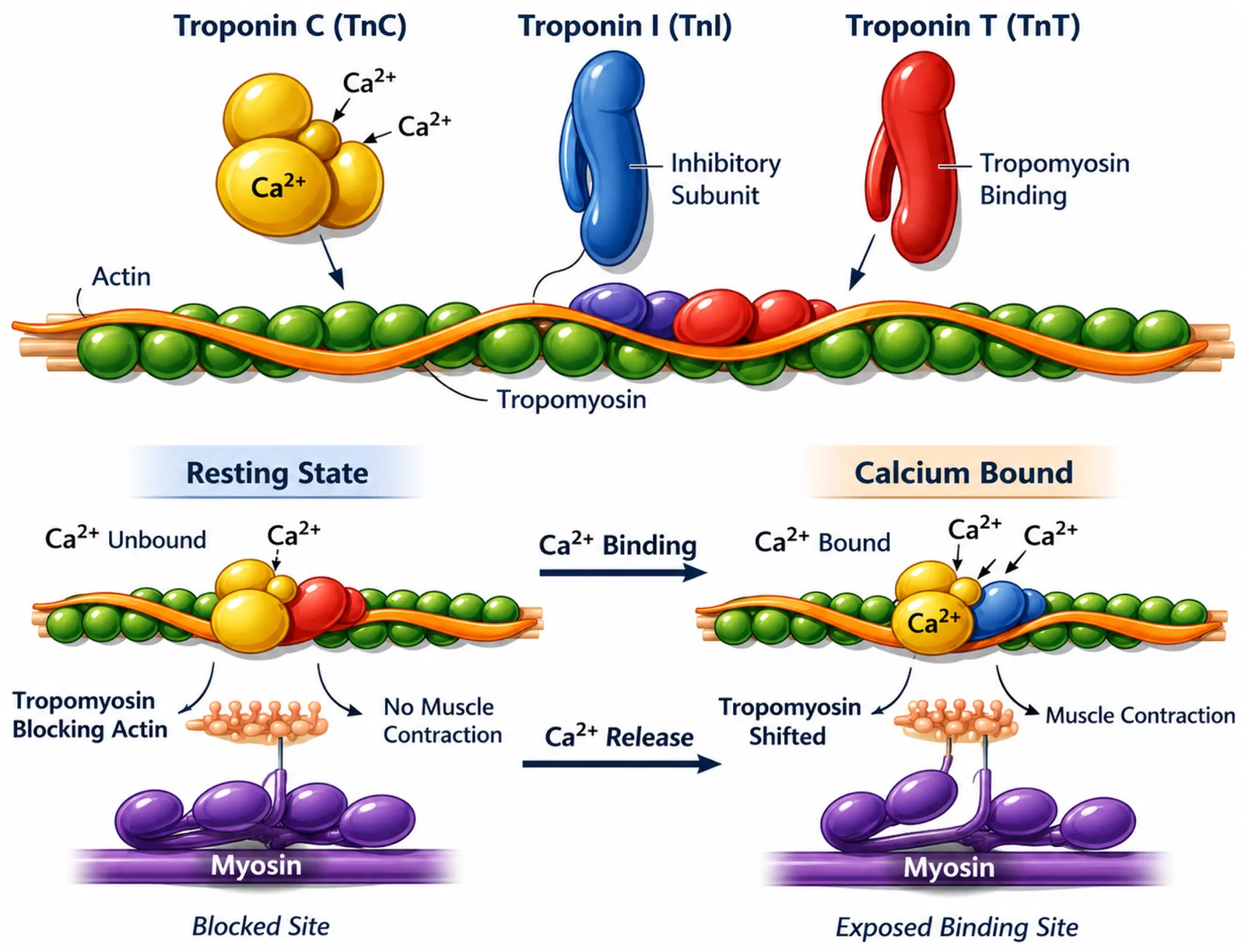
Schematic illustration of the troponin complex (TnC, TnI, and TnT) and its role in regulating muscle contraction through Ca^2+^-dependent movement of tropomyosin along actin filaments.

Clinically, cTnI and cTnT are the most commonly used biomarkers for detecting myocardial injury.^29^ Following myocardial damage, troponin molecules are released into the bloodstream, where their concentrations begin to increase within 3–6 hours, peak within 12–24 hours, and may remain elevated for several days.^30^ This prolonged elevation makes troponin a reliable marker for identifying cardiac events even when patients present later after symptom onset. Elevated troponin levels are not limited to myocardial infarction; they may also occur in other pathological conditions such as myocarditis, heart failure, pulmonary embolism, and severe systemic infections.^31^

In recent years, significant efforts have been directed toward developing advanced biosensing technologies for rapid and ultrasensitive troponin detection. Hayat *et al.* presents a portable and cost-effective microchip-based biosensor capable of detecting cTnI using only 0.5–1 mL of serum.^32^ The device utilizes a cTnI-imprinted polymer and a functionalized microchip interface to enhance specificity and performance, achieving a low limit of detection (LOD) of 0.23 ng mL^−1^ and a working range of 15.625–1000 pg mL^−1^, which includes the clinically relevant threshold for AMI diagnosis. Preliminary clinical validation demonstrated 96.8% accuracy compared to commercial assays, indicating its strong potential for rapid and minimally invasive cardiac monitoring in emergency and routine healthcare settings.^32^ Husile Bai *et al.* developed a simple enzyme-linked hybrid sandwich assay for the sensitive detection of cTnI. The study introduced a novel capture antibody–cTnI–detection aptamer configuration by combining antibody and aptamer recognition elements to enhance assay specificity and performance. The optimal antibody, aptamer interaction and the ratio between streptavidin, horseradish peroxidase (SA–HRP) and biotin-modified aptamer were systematically optimized. Interestingly, bovine serum albumin (BSA) was used not only as a blocking agent but also as a signal enhancer, improving assay sensitivity and reproducibility. The developed BSA-assisted ELHSA achieved a LOD of 0.24 ng mL^−1^ for cTnI with excellent selectivity, indicating its potential as an effective alternative to conventional ELISA-based approaches for cardiovascular disease diagnosis.^33^

Li *et al.* developed a novel aptamer-based electrochemical sensor for the highly sensitive detection of cTnI, a specific and reliable biomarker for AMI diagnosis.^34^ The sensor was fabricated using a silver nanoparticles (AgNPs)/MoS_2_/reduced graphene oxide (rGO) nanocomposite platform, where the synergistic combination of these materials enhanced conductivity, surface area, and stable aptamer immobilization. Under optimized conditions, the proposed aptasensor achieved sensitive cTnI detection over a linear range of 0.3 pg mL^−1^ to 0.2 ng mL^−1^. Moreover, excellent selectivity was demonstrated against potential interfering substances, including urea, glucose, myoglobin, dopamine, and hemoglobin, indicating its potential application for accurate and early AMI diagnosis.^34^ In another work, Gupta *et al.* developed an impedimetric electrochemical immunosensor for the rapid detection of cTnI using a copper-based metal–organic framework/polyaniline (Cu_3_(BTC)_2_/PANI) composite.^35^ The Cu_3_(BTC)_2_/PANI material was synthesized through a solvothermal approach and deposited as a conductive thin film onto screen-printed carbon electrodes (SPEs), followed by immobilization of anti-cTnI antibodies. The incorporation of PANI enhanced the electrical conductivity of the MOF-based platform, enabling sensitive impedance-based recognition of cTnI. The fabricated immunosensor detected cTnI within a clinically relevant range of 1–400 ng mL^−1^ with a rapid response time of approximately 5 min. Furthermore, high selectivity toward cTnI was demonstrated even in the presence of potentially interfering biomolecules, indicating its potential for fast cardiac biomarker diagnosis.^35^ The aforementioned methods rely on different materials, some of which may not be low-cost and can be difficult to prepare. In addition, the availability and processing requirements of these materials may limit the practicality and large-scale implementation of the proposed approaches.^36^ An emerging class of nanomaterial-based biosensors is CD-based platforms, which have been increasingly explored for cardiac troponin detection due to the unique physicochemical and optical properties of CDs, including excellent fluorescence behavior, high biocompatibility, water solubility, tunable surface functionalities, and low toxicity.^37^ Carbon dots possess unique optical and chemical properties, including strong fluorescence, high photostability, excellent biocompatibility, and facile surface functionalization. These characteristics make CDs promising nanoprobes for the ultrasensitive detection of cardiac biomarkers such as troponin, offering new opportunities for early diagnosis and improved management of cardiovascular diseases.^24^ Additionally, current challenges, future perspectives, and the potential clinical applicability of CDs-based biosensing technologies for next-generation cardiovascular diagnostics are critically discussed.

## 3. Carbon dots (CDs)

CDs are carbon nanomaterials with particle sizes smaller than 10 nm and have attracted significant attention because of their unique physicochemical and photochemical properties.^38^ These features make them useful in many fields such as bioimaging, biodetection, catalysis, environmental applications, light-emitting devices, and agriculture.^39^ Although their applications have grown rapidly, understanding their exact physicochemical properties remains challenging due to their complex structure.^40^ CDs exhibit excellent luminescence properties, including high quantum yield, strong photostability, long phosphorescence lifetime, and a broad emission wavelength range, making them promising for optical and optoelectronic applications.^15^ However, understanding their luminescence mechanisms and structure–property relationships remain challenging due to their complex structures and synthesis processes.^41^ CDs are small carbon nanomaterials that have attracted significant attention because of their unique properties and wide range of applications. Recent studies show that CDs can consist of different fractions and can also serve as building blocks for larger carbon-based materials.^42^ Understanding the relationship between their structure, properties, and activity is important for improving their performance and expanding their use in fields such as photochemistry, electrochemistry, nanomedicine, and materials science.^43^ CDs have attracted growing interest because of their low toxicity, chemical stability, tunable fluorescence, good water solubility, and favorable physicochemical properties.^44^ These characteristics make them useful in applications such as chemical and biological sensing, fluorescence imaging, and drug delivery. Recent studies have also focused on green synthesis methods and the possible luminescence mechanisms of CDs, including surface states, quantum confinement effects, and molecular fluorescence, as well as their applications in both solution-phase and solid-phase fluorescence sensing.^45^

The luminescence properties of CDs are strongly influenced by their size, morphology, surface functional groups, degree of carbonization, heteroatom composition, and the nature of their electronic and surface states.^46,47^ In particular, surface defects and emissive sites can significantly affect fluorescence intensity, emission wavelength, quantum yield, and photostability, while changes in pH, solvent polarity, ionic strength, temperature, and the surrounding chemical environment may further modulate their optical response. Structural and chemical engineering strategies, especially heteroatom doping with elements such as nitrogen, sulfur, phosphorus, and boron, have been widely employed to tailor the electronic structure and introduce additional emissive states, thereby enhancing fluorescence efficiency and extending the emission range. Co-doping with two or more heteroatoms can provide synergistic effects and further improve optical and sensing performance.^48^ Owing to their tunable photoluminescence, excellent water dispersibility, low toxicity, good photostability, and facile surface functionalization, CDs have attracted considerable attention in a wide range of applications, including chemical and biosensing, fluorescence imaging, environmental monitoring, photocatalysis, light-emitting devices, energy conversion, and analytical detection of biologically relevant species. In sensing applications, their luminescence can be modulated through mechanisms such as fluorescence quenching, enhancement, Förster resonance energy transfer, inner-filter effects, and aggregation-induced changes, making CDs particularly promising as versatile optical probes.

### 3.1. Synthesis of carbon dots

CDs can be synthesized through different methods, mainly classified as top-down and bottom-up approaches as shown in Scheme 2. In recent studies, green synthesis using renewable and natural precursors has gained significant attention because it is simple, cost-effective, and environmentally friendly. One-pot green synthesis methods avoid the use of harsh chemicals and energy-intensive processes, making them safer and more sustainable. However, challenges remain in controlling the size, structure, and optical properties of carbon dots, particularly their fluorescence quantum yield, which requires further research to improve their performance and broaden their applications.^49^

**Scheme 2:**
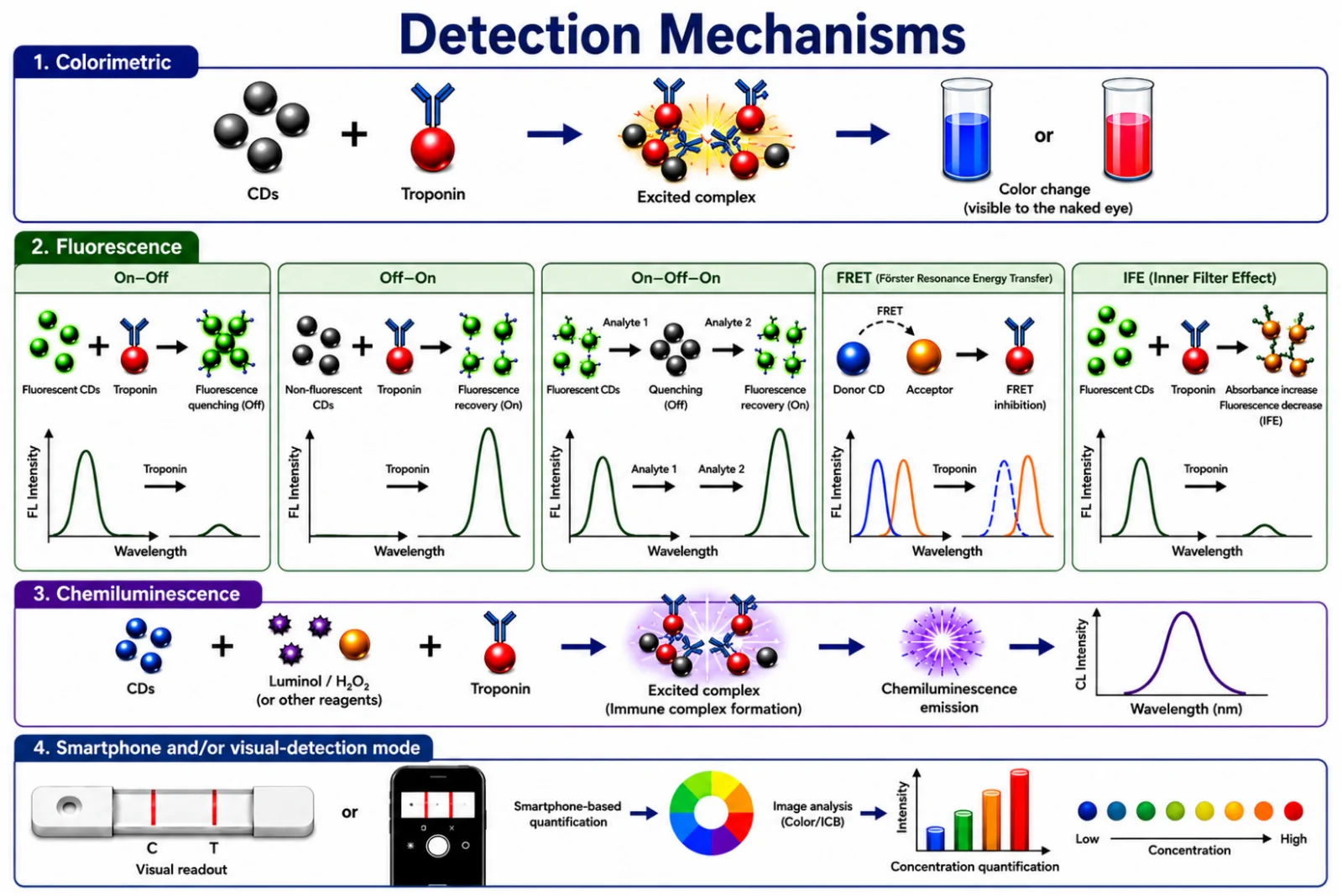
Schematic representation of CD-based troponin detection mechanisms. The figure illustrates colorimetric, fluorescence (on–off, off–on, FRET, and IFE), chemiluminescence, and smartphone-assisted sensing strategies, where signal changes produced by CD–troponin interactions enable sensitive and rapid cardiac biomarker detection.

#### 3.1.1 Bottom-up approach

The bottom-up approach is one of the most widely used strategies for synthesizing CDs. In this method, CDs are formed from small molecular precursors such as organic molecules, carbohydrates, polymers, or biomass through processes including dehydration, polymerization, and carbonization, which produce nanoscale carbon particles with tunable optical and surface properties.^50^ Compared with top-down methods, bottom-up synthesis offers advantages such as simple procedures, low cost, environmentally friendly precursors, and better control over particle size and surface functional groups.^51^ Additionally, heteroatom doping with elements such as nitrogen, sulfur, or phosphorus can be introduced during synthesis to enhance fluorescence and sensing performance. Common bottom-up techniques include hydrothermal or solvothermal synthesis, where precursors such as citric acid are heated in sealed autoclaves to form fluorescent CDs.^52^ Another widely used method is microwave-assisted synthesis, which provides rapid and uniform heating, significantly reducing reaction time and improving production efficiency.^52^ In addition, thermal pyrolysis involves the thermal decomposition of organic precursors at elevated temperatures to generate highly luminescent carbon nanoparticles.^53^ Other bottom-up strategies, including electrochemical and combustion synthesis, have also been reported to tailor the structure and optical properties of CDs for applications in sensing, bioimaging, catalysis, and environmental.

#### 3.1.2 Top-down approach

The top-down approach is another common strategy for synthesizing CDs as shown in Scheme 2, in which larger carbon structures such as graphite, carbon nanotubes, graphene, carbon fibers, or soot are broken down into nanoscale carbon particles through physical or chemical processes.^54^ This method typically involves techniques such as laser ablation, arc-discharge, chemical oxidation, and electrochemical exfoliation, which break bulk carbon materials into small fluorescent carbon nanoparticles.^50^ Compared with bottom-up synthesis, top-down methods are advantageous because they utilize existing carbon frameworks and can produce CDs with relatively high crystallinity and stable structures.^54^ For example, laser ablation involves irradiating carbon targets with high-energy laser pulses to generate nanosized carbon particles, while chemical oxidation uses strong oxidizing agents (*e.g.*, nitric acid or sulfuric acid) to cut larger carbon materials into carbon dots with oxygen-containing functional groups on their surfaces.^55^ Similarly, electrochemical synthesis produces CDs by electrochemical exfoliation of graphite electrodes in suitable electrolytes.^55^ However, top-down approaches often require harsh reaction conditions, expensive equipment, or complex purification steps, which may limit large-scale production. Despite these limitations, top-down synthesized CDs exhibit excellent optical properties and remain widely used in applications such as bioimaging, sensing, catalysis, and optoelectronic devices.^54^Fig. 2 shows the two main approach of CDs synthesis. In the top-down route, large bulk carbon materials are broken down into nanoscale carbon dots using methods such as electrochemical exfoliation, laser ablation, and chemical oxidation. In contrast, the bottom-up approach involves the formation of CDs from small molecular precursors through carbonization processes, including microwave irradiation, hydrothermal, solvothermal, and ionothermal synthesis. During synthesis, carbonization forms the carbon core, while surface passivation modifies surface functional groups, improving the optical, electronic, and chemical properties of CDs for various sensing and biomedical applications.

**Fig. 2:**
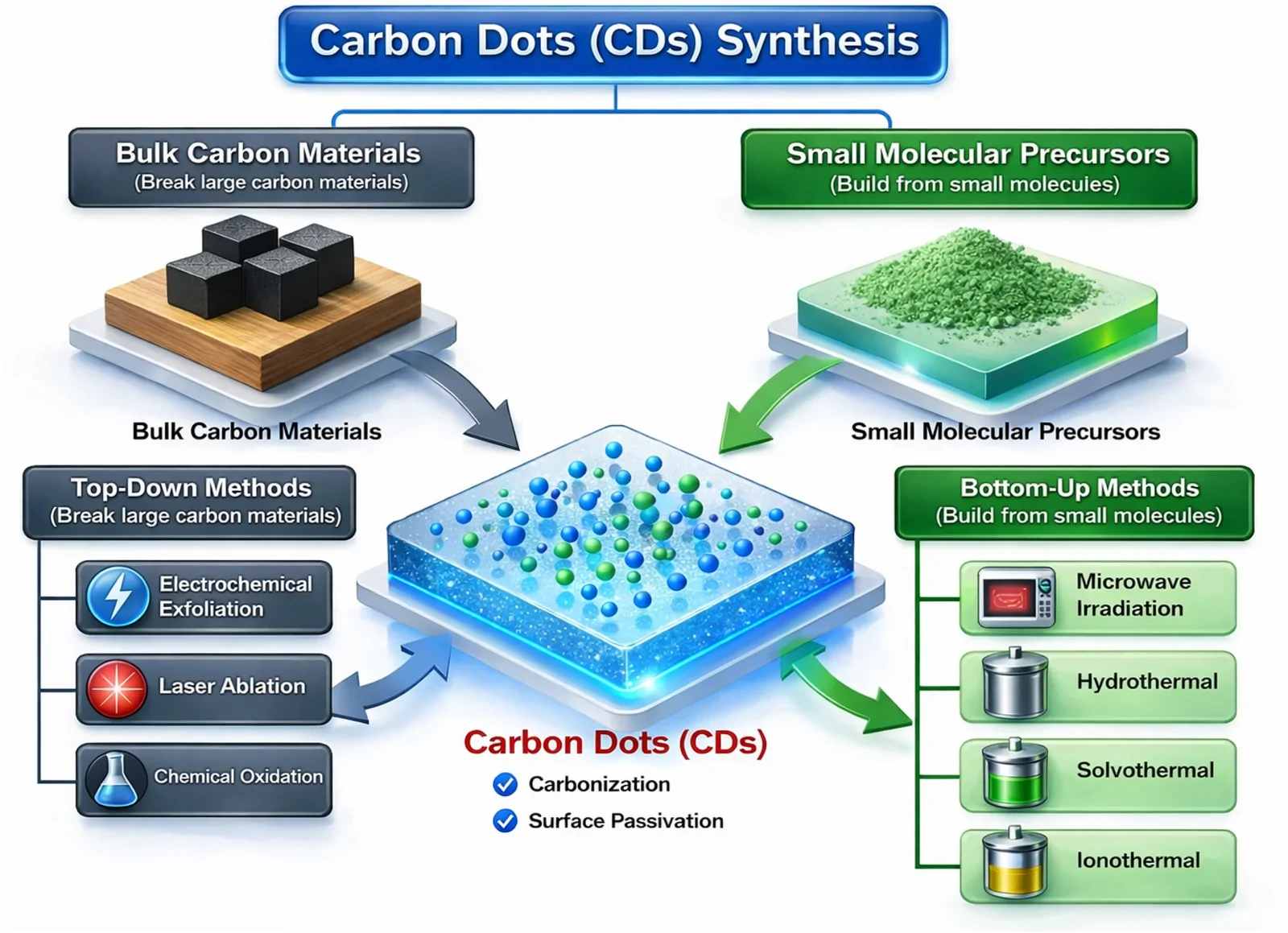
Representation of CDs synthesis *via* top-down and bottom-up approaches. Bulk carbon sources are converted into CDs through exfoliation, ablation, or oxidation, whereas small molecular precursors undergo carbonization *via* hydrothermal, solvothermal, or microwave-assisted routes, followed by surface passivation.

## 4. Detection mechanisms

Various detection strategies have been developed and widely employed for troponin analysis in analytical chemistry, including colorimetric, fluorescence, and chemiluminescence-based methods. Each of these approaches relies on distinct signal-generation mechanisms and offers specific advantages in terms of sensitivity, selectivity, simplicity, and instrumentation requirements. However, they also present inherent limitations, such as assay complexity, susceptibility to matrix interference, relatively high instrumentation costs, or limited applicability in certain analytical settings. Consequently, the selection of an appropriate detection mechanism depends on the specific requirements of the troponin assay, including the desired sensitivity, accuracy, response time, and suitability for point-of-care or laboratory-based applications.

### 4.1. Colorimetric detection

Colorimetric detection is based on visible color changes that occur upon the interaction between CD-based sensing platforms and cardiac troponins. In these systems, CDs may act either as nanozymes, signal amplifiers, or catalytic mediators that induce changes in the absorbance properties of chromogenic substrates. The binding of troponin to recognition elements such as antibodies or aptamers alters the optical response, resulting in a detectable color transition that can often be observed by the naked eye. Due to its simplicity, low cost, and rapid response, colorimetric sensing is considered attractive for point-of-care and resource-limited diagnostic applications.

### 4.2. Fluorescence detection mechanism

Is the most widely explored mechanism in CD-based biosensors because of the exceptional photoluminescence properties of carbon dots, including high quantum yield, tunable emission, and excellent photostability. In these systems, the fluorescence intensity of CDs changes after interaction with cardiac troponins through several sensing modes, including “on–off,” “off–on,” and “on–off–on” mechanisms. Fluorescence quenching or recovery may occur due to electron transfer, surface passivation, or analyte-induced structural changes. More advanced fluorescence mechanisms include FRET, where energy transfer occurs between donor and acceptor fluorophores, and the Inner Filter Effect (IFE), in which absorption overlap suppresses fluorescence emission. These fluorescence strategies enable highly sensitive and selective detection of cardiac troponins at very low concentrations.

### 4.3. Chemiluminescence detection mechanism

Chemiluminescence-based detection relies on the emission of light generated from chemical reactions involving CDs and luminescent reagents such as luminol or hydrogen peroxide. In CD-assisted chemiluminescent biosensors, carbon dots can function as catalysts, electron-transfer mediators, or signal enhancers that improve light emission efficiency during troponin recognition. The formation of antigen–antibody or aptamer–troponin complexes modulate the chemiluminescent intensity, which is quantitatively correlated with troponin concentration. Compared with conventional optical techniques, chemiluminescence offers high sensitivity, low background interference, and excellent signal amplification, making it promising for ultrasensitive cardiac biomarker detection.

### 4.4. Smartphone-based and visual detection mechanism

Smartphone-based and visual detection approaches have recently emerged as attractive strategies for cardiac troponin sensing due to their portability, simplicity, and suitability for point-of-care testing. In these systems, CDs are employed as optical probes that generate detectable colorimetric or fluorescent signals in the presence of troponins, while smartphones function as portable analytical devices for signal acquisition and processing. The captured images are analyzed using smartphone cameras or dedicated applications to quantify color intensity, fluorescence changes, or RGB values, enabling rapid and user-friendly diagnostics without sophisticated instrumentation. Visual-based sensing platforms also allow semi-quantitative or qualitative detection through naked-eye observation of color or fluorescence changes. Detection mechanisms of CD-based troponin sensors involve colorimetric, fluorescence, chemiluminescence, and smartphone-assisted approaches. These strategies rely on signal changes caused by CD–troponin interactions, enabling rapid, sensitive, and portable cardiac biomarker detection show in Scheme 2.

## 5. Applications

CDs-based optical biosensors have attracted considerable attention for cardiac troponin detection owing to their exceptional photoluminescence properties, excellent photostability, high sensitivity, low toxicity, and flexible surface functionalization capabilities.^54^ These unique characteristics enable CDs to act as efficient fluorescent probes, optical signal transducers, and sensing platforms for the selective recognition of cardiac biomarkers such as cardiac troponins.^55^ In particular, CD-based biosensors offer significant advantages for early diagnosis and point-of-care (POC) monitoring by providing rapid response, simple operation, and enhanced detection performance.^56^ In recent years, various CD-assisted optical sensing strategies have been developed for ultrasensitive troponin detection, with fluorescence, colorimetric, and chemiluminescence-based approaches representing the most widely explored techniques (Table 2).^56^ Fluorescence-based platforms utilize variations in emission intensity, wavelength shifts, or fluorescence quenching/recovery mechanisms to accurately quantify troponin concentrations with high sensitivity.^55^ Meanwhile, colorimetric and chemiluminescent systems provide alternative detection pathways through visible signal changes or amplified light-generation processes, enabling rapid and reliable biomarker detection in clinical and analytical applications.^57^ The integration of CDs with specific biomolecular recognition elements, including antibodies and aptamers, further enhances selectivity and allows accurate detection of cardiac troponins in complex biological samples.^57^ Therefore, CD-based optical biosensors represent promising next-generation platforms for rapid, sensitive, and cost-effective cardiac biomarker analysis in clinical diagnostics.

**Table 2:** Comparative analytical and clinical characteristics of carbon-dot- and quantum-dot-based sensing platforms for cardiac troponin detection, including recognition elements, sample types, response times, linear ranges, limits of detection, clinical validation, advantages, and limitations^a^

Material	Method	Recognition element	Sample type	Response time	Linear range (ng mL^−1^)	LOD (ng mL^−1^)	Clinical validation	Advantages	Limitations	Ref.
CDs/MoS_2_	FL (FRET)	Anti-cTnT antibody	Serum application reported	NR	0.1–50	0.12	Analytical serum application; patient-cohort validation and comparison with a clinical reference assay NR	Turn-on FRET response; antibody-based selectivity	Response time, patient-sample agreement and inter-batch precision NR	58
CdSe/ZnS QDs–SPION	FL (turn-on)	Tro4 aptamer	Aqueous buffer	30 min	2–40	13.3	Not performed	Magnetically recoverable; reusable for three cycles; stable for 30 days at 4 °C	Relatively high LOD; Cd-containing QDs; no patient-serum validation; pH-sensitive fluorescence	59
GO/GCE–N,Zn-GQDs	Ratiometric ECL/DPV	Anti-cTnI antibody	Human serum	NR	0.00001–1	0.00000459	Preliminary method comparison with hospital ELISA; RSD 9.09–11.1%	Ultralow LOD; unlabeled ratiometric dual-signal readout; serum applicability	Requires electrochemical/ECL instrumentation; response time, patient-cohort size and inter-batch precision NR	60
Au/fGQDs	Electrochemical (CV/amperometry)	Antibody-free direct cTnI–fGQD interaction	0.1 M NaCl; purified human cTnI	10 s	0.17–3	0.02	Bland–Altman agreement with calibrated assay; no patient-sample validation	Rapid response; antibody-free; RSD 3.1% (*n* = 3); low LOD	Narrow range; trypsin also generated peaks at different potentials; clinical validation in a substantial patient cohort required	61
anti-cTnT–caf-TCDs/GO	FL (turn-on FRET)	Monoclonal anti-cTnT antibody	Buffer and serum	NR	0.05–50	0.046	Analytical testing in serum; patient cohort and clinical-reference comparison NR	Wide range; fluorescence recovery; high specificity; carbodiimide-linked immunoprobe	Quantitative response time, serum-sample details, reproducibility and clinical validation NR	62
caf-TCQDs@AuNPs/BNNS	Electrochemical	NR	NR	NR	0.0001–100*	0.0013	NR	Nanocomposite electrode interface; broad reported analytical range	Recognition element, matrix, response time, reproducibility, clinical validation, and starred analytical values require full-text verification	63
MnO_2_/MPA–Mn^2+^: ZnS QDs	FL (turn-on)	NR	NR	NR	0–2	0.05	NR	Turn-on fluorescence based on MnO_2_-mediated QD quenching	Recognition element, matrix, response time, reproducibility, clinical validation, and starred analytical values require full-text verification	64
CdSe/CdS/ZnS QDs/TiO_2_	PEC	Anti-cTnI antibody	cTnI standard (clinical specimens NR)	NR	0.01–0.2	0.01	No clinical validation reported	Enhanced charge separation, photocurrent and chemical stability	Narrow detection range; response time, reproducibility and patient-sample performance not reported	65
DA–AuNP–GQ, D–MIP	Electrochemical (CV/SWV/EIS)	cTnI epitope-imprinted polydopamine	Human serum (spiked recovery)	NR	0.01–20	NR	Recovery in spiked human serum; no prospective patient-cohort validation	Antibody-free recognition; maximum imprinting factor 5.9; high selectivity	LOD and response time not reported in the abstract; clinical agreement and sensor-to-sensor reproducibility NR	66

a CDs, carbon dots; CQDs, carbon quantum dots; FRET, Förster resonance energy transfer; FL, fluorescence intensity; ECL, electrochemiluminescence; PEC, photoelectrochemical; AuNPs, gold nanoparticles; GODs, graphene oxide dots; MoS_2_, molybdenum disulfid

As summarized in Table 2, CD-based biosensors have been widely developed for the sensitive detection of cardiac troponin (cTnI) using different signal transduction strategies, including fluorescence, electrochemiluminescence (ECL), photoelectrochemical (PEC), and electrochemical methods. According to the comparative analysis, ECL-based platforms demonstrate the highest analytical sensitivity among the reported approaches. In particular, caf-TCQDs@AuNPs achieved an ultra-low LOD of 0.0013 ng mL^−1^ with a broad linear range of 0.0001–100 ng mL^−1^, while N/Zn-CQDs provided a wider detection range of 0.01–1000 ng mL^−1^ with a low LOD of 0.00459 ng mL^−1^. These results highlight the strong potential of ECL-based CQD platforms for early and highly sensitive cTnI detection. Fluorescence-based biosensors also showed effective detection capability with simple operation and reliable optical responses. CDs/MoS_2_ and Mn^2+^-doped ZnS-CQDs achieved LOD values of 0.12 and 0.05 ng mL^−1^, respectively, indicating good sensitivity for cTnI monitoring. Furthermore, caf-TCDs exhibited improved fluorescence performance with an LOD of 0.046 ng mL^−1^ across a wide detection range of 0.05–50 ng mL^−1^. However, CdSe/ZnS-CQDs showed comparatively lower sensitivity, with a higher LOD of 13.3 ng mL^−1^.

Other sensing strategies, including PEC and electrochemical approaches, provide alternative platforms for cTnI detection. CdSe/CdS/ZnS CQDs-based PEC sensors displayed a detection range of 0.2–10 ng mL^−1^ with an LOD of 10 ng mL^−1^, whereas AuNPs/GQDs electrochemical sensors achieved higher sensitivity with an LOD of 0.01 ng mL^−1^ over a range of 0.01–20 ng mL^−1^. Overall, ECL-based CQD biosensors offer the best detection performance in terms of sensitivity and linear range, while fluorescence and electrochemical systems remain promising for rapid, portable, and point-of-care cardiac biomarker analysis.

### 5.1. Fluorescence-based applications

Fluorescence-based CDs biosensors are among the most extensively investigated optical sensing platforms for cardiac troponin detection due to their strong photoluminescence, high sensitivity, excellent stability, and tunable emission properties.^50^ These biosensors mainly rely on changes in fluorescence signals, including fluorescence quenching, enhancement, or recovery, after specific interactions between CDs and cardiac biomarkers such as troponin or antibody–antigen complexes.^67^ The adjustable optical characteristics of CDs allow precise signal modulation, which is particularly important for detecting extremely low biomarker concentrations associated with early-stage myocardial infarction.^62^

Another fluorescence-based approach was reported by Zhang *et al.*, who designed a carbon dot immunosensor using carboxyl-functionalized triangular carbon dots (caf-TCDs) for cTnT detection.^58^ In this system, the strong interaction between CDs and GO caused significant fluorescence suppression. After the addition of cTnT, the formation of antigen–antibody immune complexes weakened the CD–GO interaction, preventing energy transfer and restoring the fluorescence signal. The developed biosensor exhibited a wide linear detection range of 0.05–50 ng mL^−1^ and a low detection limit of 0.046 ng mL^−1^, confirming the high sensitivity of CDs-based fluorescence platforms for cardiac biomarker detection.^58^ As shown in Fig. 3A, the sensing mechanism involves the synthesis of CDs from carbohydrate precursors through carbonization, followed by antibody functionalization using EDC/NHS coupling chemistry.^58^ Under near-infrared (NIR) excitation (900–950 nm), antibody-modified CDs generate green up-conversion fluorescence. When interacting with two-dimensional nanosheets such as GO or MoS_2_, fluorescence quenching occurs through the FRET process. However, in the presence of cTnT, specific antigen–antibody binding produces an immune complex that separates CDs from the quencher surface, suppresses FRET efficiency, and restores fluorescence emission, allowing sensitive quantitative detection of cardiac troponin.^58^ In another work, Mishra *et al.* developed a cardiac immunosensor based on carboxyl-functionalized triangular carbon dots (caf-TCDs) conjugated with anti-cTnT and GO for early myocardial infarction detection. The sensing mechanism relied on GO-induced fluorescence quenching through energy transfer, followed by fluorescence recovery after specific cTnT–anti-cTnT binding. The sensor exhibited high selectivity toward cTnT with a linear detection range of 0.05–50 ng mL^−1^ and a low detection limit of 0.046 ng mL^−1^, demonstrating its potential for sensitive cardiac biomarker detection in biological samples.^62^ CD-based and nanomaterial-assisted optical biosensors have demonstrated excellent potential for cardiac troponin detection due to their strong fluorescence response, high sensitivity, and efficient signal amplification. The integration of CDs with advanced nanomaterials enables fluorescence recovery, FRET modulation, colorimetric sensing, and chemiluminescence detection mechanisms. Representative optical sensing strategies for cardiac troponin detection are shown in Fig. 3B.^62^

**Fig. 3:**
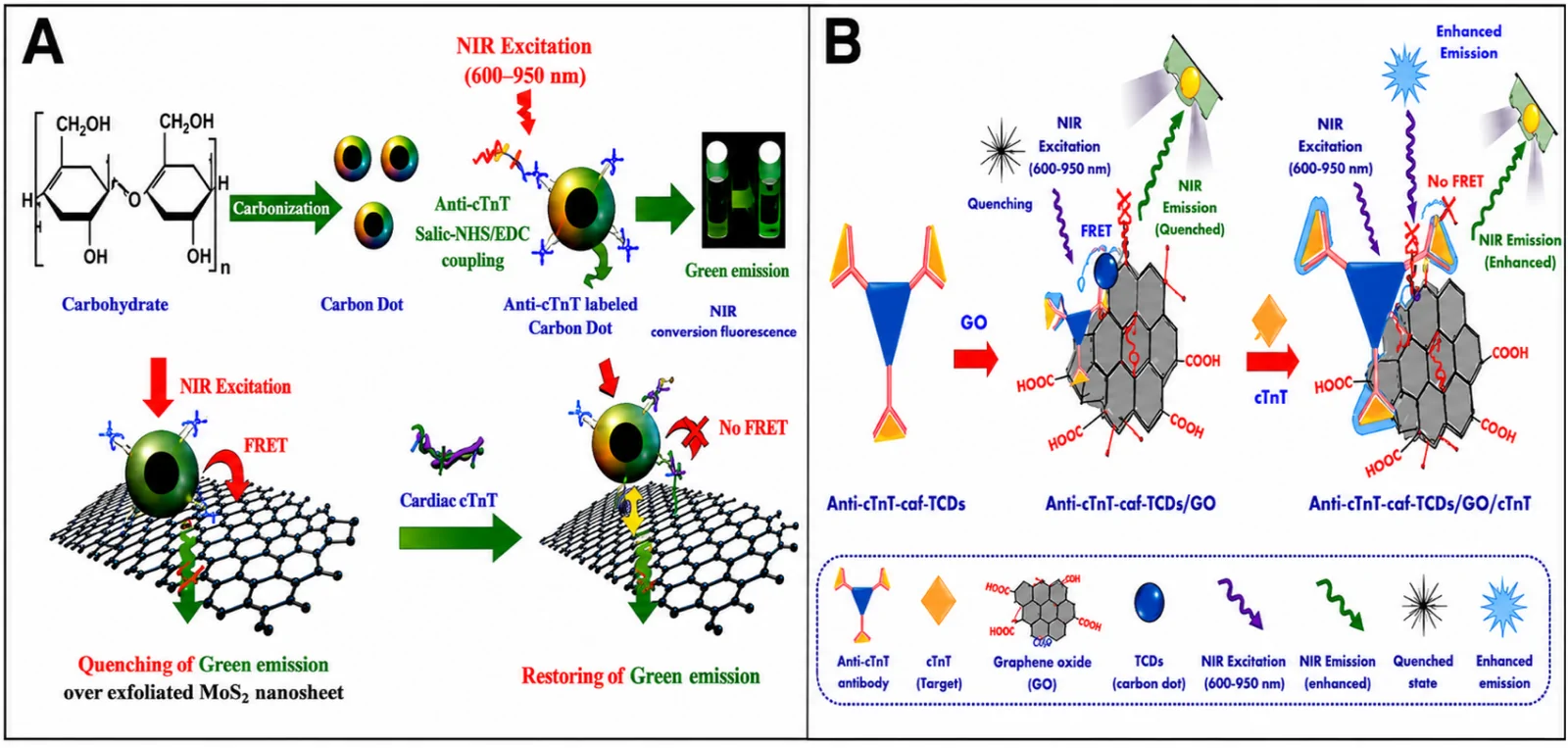
CD-based and nanomaterial-assisted optical biosensing strategies for cardiac troponin detection. (A) Fluorescence-based detection mechanism using anti-cTnT-functionalized carbon dots, where fluorescence recovery occurs through specific antigen recognition and inhibition of energy transfer interactions. Reprinted with permission form ref. 58. (B) FRET-based immunosensing system using graphene oxide/nanomaterial-assisted fluorescence quenching and recovery for sensitive cardiac troponin detection. Reprinted with permission form ref. 62.

### 5.2. Colorimetric-based applications

Colorimetric CD-based biosensors enable cardiac troponin detection through visible color changes produced by catalytic reactions, biochemical interactions, or nanoparticle aggregation processes. These optical responses allow rapid and simple analysis without the requirement for sophisticated laboratory instruments.^58^ Therefore, colorimetric sensing platforms are highly attractive for point-of-care testing and applications in resource-limited environments due to their advantages of low cost, easy operation, and visual signal interpretation.^68^ These biosensors have been widely incorporated into lateral flow assays, paper-based analytical devices, and portable diagnostic platforms for rapid bedside cardiac biomarker monitoring.^69^ Furthermore, dual-mode sensing approaches combining colorimetric and fluorescence responses have been developed to enhance analytical reliability, sensitivity, and detection.^70^

A gold nanoparticle-based colorimetric assay was developed for rapid detection of cTnI using a specific aptamer as the molecular recognition element.^71^ The sensing mechanism is based on aptamer-mediated regulation of salt-induced gold nanoparticle aggregation, where interaction between cTnI and the aptamer produces a visible colorimetric response. Under optimized experimental conditions (200 mM NaCl and 800 nM aptamer), the platform exhibited a linear detection range of 0.1–0.8 µg mL^−1^ (*R*^2^ = 0.942) with a detection limit of 0.1 µg mL^−1^ and recovery values ranging from 105.6% to 118.5%. These findings demonstrate the applicability of this approach as a simple, sensitive, and low-cost strategy for point-of-care myocardial infarction assessment.^71^

Ulloa-Gomez *et al.* developed a microfluidic paper-based analytical device (µ-PAD) aptasensing platform for cTnT detection using gold-decorated polystyrene microparticles functionalized with a specific aptamer.^72^ The proposed colorimetric system showed two linear detection ranges (0.01–0.8 µg mL^−1^ and 6.25–50 µg mL^−1^) with a low LOD of 3.9 × 10^−4^ µg mL^−1^. In addition, the sensor demonstrated excellent selectivity against other cardiac biomarkers, smartphone-assisted quantitative analysis capability, and good stability for 23 days, indicating its suitability as a portable and cost-effective point-of-care diagnostic platform (Fig. 4B).^72^

**Fig. 4:**
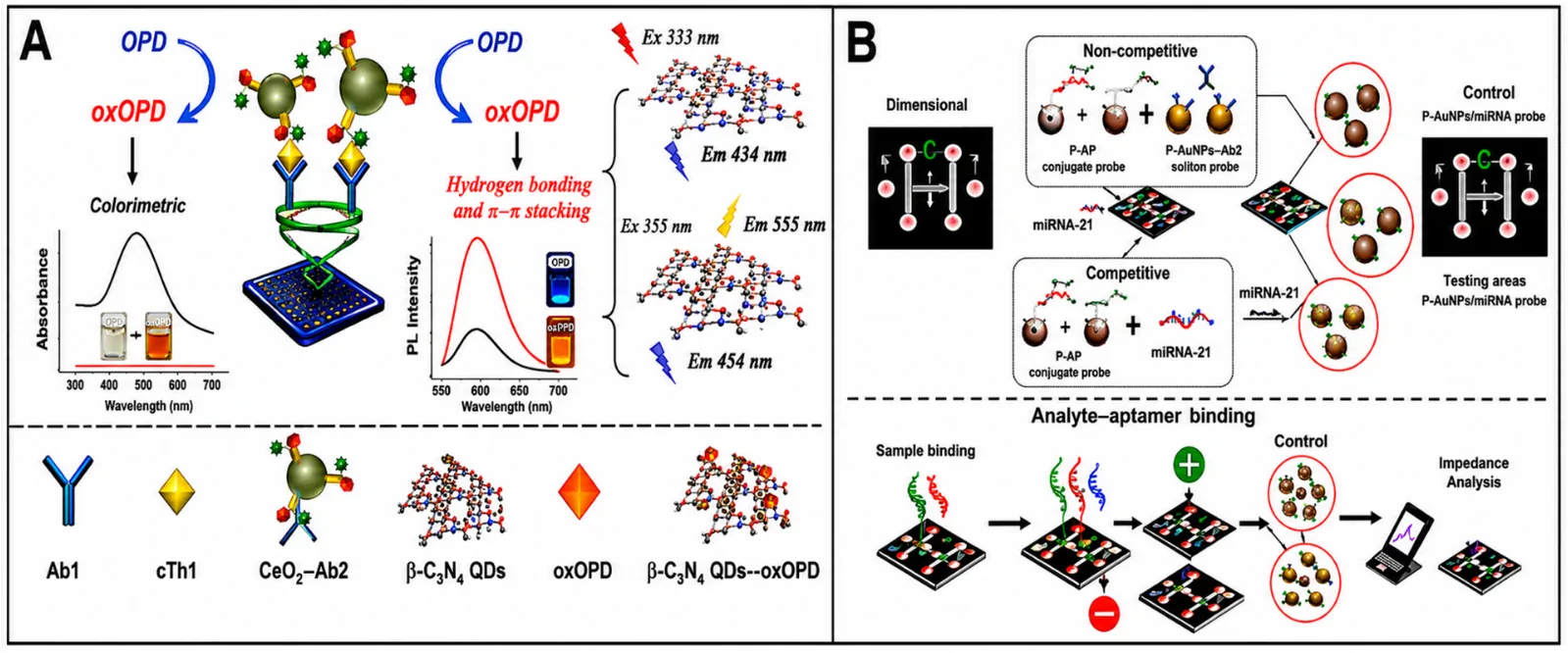
(A) Dual-mode colorimetric and ratiometric fluorescence immunosensor based on CeO_2_ nanozyme catalytic activity and β-C_3_N_4_ quantum dots for cTnI detection through OPD oxidation and fluorescence modulation. Reprinted with permission form ref. 75. (B) Three-dimensional microfluidic paper-based chemiluminescence immunoassay utilizing functionalized magnetic carbon composites for multiplex detection of AMIbiomarkers. Reprinted with permission form ref. 72.

Colorimetric biosensors detect troponin through visible optical variations caused by biochemical reactions or nanoparticle aggregation. These color changes can be directly observed by the naked eye or quantitatively analyzed using simple spectrophotometric methods.^73^ In several sensing designs, carbon dots are combined with catalytic nanomaterials or metallic nanoparticles to improve signal amplification and analytical performance. These properties make colorimetric biosensors promising candidates for rapid, affordable, and portable cardiac biomarker detection.^74^

For example, Miao *et al.* developed a nanozyme-linked immunosorbent assay for dual-modal cTnI detection by integrating ratiometric fluorescence and colorimetric sensing.^75^ In this system, nanoceria with peroxidase-like activity catalyzes the oxidation of *o*-phenylenediamine (OPD) into 2,3-diaminophenazine (oxOPD) in the presence of H_2_O_2_, generating a visible colorimetric signal. Meanwhile, the produced oxOPD interacts with graphitic carbon nitride quantum dots (g-C_3_N_4_ QDs) through hydrogen bonding and π–π interactions, leading to fluorescence quenching at 434 nm and the formation of a new emission peak at 556 nm. The combination of fluorescence and colorimetric responses enhances detection sensitivity, accuracy, and reliability. As illustrated in Fig. 4A, this dual-mode sensing strategy provides both visual colorimetric detection and fluorescence-based quantitative analysis, demonstrating strong potential for advanced cardiac biomarker sensing applications.^75^

### 5.3. Chemiluminescence-based applications

Chemiluminescence-based CD biosensors have emerged as highly sensitive optical platforms for cardiac troponin detection due to their ability to generate light signals through chemical reactions without external excitation sources.^76^ The absence of excitation light significantly reduces background interference and improves signal-to-noise ratios, allowing ultra-sensitive detection of low-abundance biomarkers associated with early-stage cardiovascular diseases.^77^ Owing to their excellent sensitivity, wide dynamic range, and signal amplification capability, chemiluminescence-based sensing systems have been extensively applied in high-performance immunoassays and lateral flow platforms for cardiac biomarker analysis.^78^ Moreover, their compatibility with automated diagnostic instruments provides improved precision, reproducibility, and reliability for clinical applications.^79^

Yang *et al.* developed a chemiluminescence immunoassay based on Co^2+^-ABEI-functionalized magnetic carbon composites integrated with a three-dimensional microfluidic paper-based analytical device (3D µPAD) for simultaneous detection of AMI biomarkers, including copeptin, heart-type fatty acid-binding protein (h-FABP), and cTnI.^80^ The sensing mechanism relies on the formation of immunocomplexes containing Co^2+^-ABEI magnetic carbon composites, which enhance chemiluminescent signal generation through catalytic amplification reactions (Fig. 5B). The 3D µPAD platform enables controlled reagent transport and time-resolved signal collection from multiple detection zones, allowing multiplex biomarker analysis. Under optimized conditions, the system achieved detection limits of 0.40 pg mL^−1^, 0.32 pg mL^−1^, and 0.50 pg mL^−1^ for copeptin, h-FABP, and cTnI, respectively. The platform exhibited excellent sensitivity, selectivity, and successful detection performance in human serum samples, demonstrating its potential for rapid and early diagnosis of AMI.^80^

**Fig. 5:**
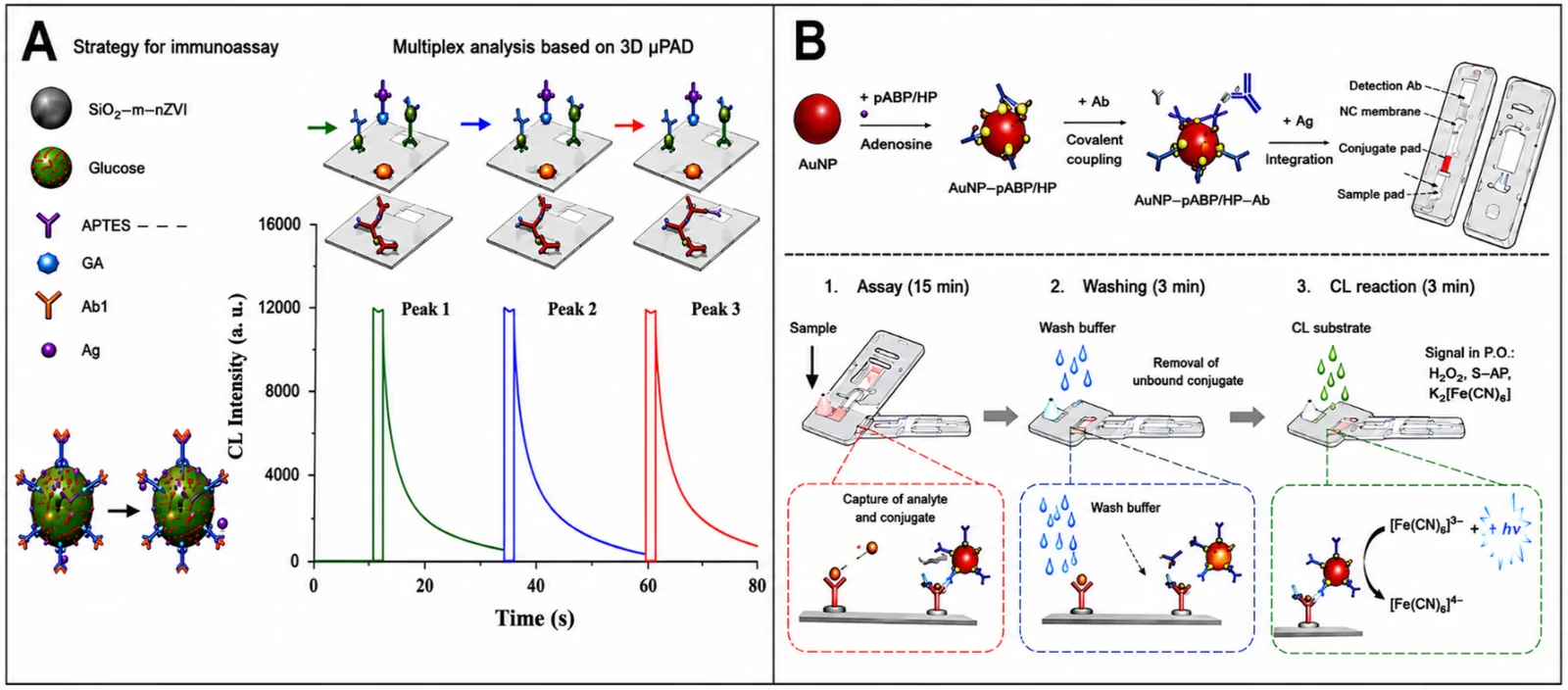
Representative chemiluminescence biosensing platforms for cardiac biomarker detection. (A) Multiplex chemiluminescence immunoassay based on a 3D µPAD. Adapted under the terms and conditions of the Creative Commons Attribution (CC BY).^57^. (B) Chemiluminescence-based lateral-flow immunoassay employing AuNP–pABP/HP–antibody conjugates for highly sensitive cTnI detection. Reprinted with permission from ref. 80.

Chemiluminescence is an optical sensing approach in which photon emission occurs as a result of chemical reactions rather than external light stimulation, thereby minimizing autofluorescence and optical background interference.^81^ In cardiac biomarker analysis, chemiluminescent immunoassays have been widely employed because of their superior analytical sensitivity and reliable quantitative performance. In CD-related sensing systems, carbon-based nanomaterials can function as catalytic components, nanocarriers, or signal-enhancement platforms to amplify chemiluminescence intensity and improve detection capability.^82^

For example, Lee *et al.* developed a highly sensitive chemiluminescence-based lateral flow immunoassay (LFA) for cTnI detection to overcome the limited sensitivity of conventional AuNP-based colorimetric assays.^57^ In this strategy, AuNPs were conjugated with aldehyde-activated HRP and specific antibodies to form AuNP-(ald)HRP-Ab signal probes. During detection, cTnI forms antigen–antibody complexes on the LFA test line, followed by HRP-mediated catalysis of luminol/H_2_O_2_ reactions to generate strong chemiluminescent emission (Fig. 5A). Compared with traditional AuNP-based colorimetric LFAs, the proposed CL-LFA achieved approximately 110-fold enhancement in detection sensitivity. The assay demonstrated a low detection limit of 5.6 pg mL^−1^ for cTnI with excellent analytical precision (coefficient of variation: 2.3–8.4%). Furthermore, analysis of clinical serum samples showed strong agreement with standard biochemical analyzers (correlation coefficient *r* = 0.97), confirming its potential as a highly sensitive point-of-care diagnostic platform for cardiac biomarker detection.^57^

## 6. Challenges and limitations

Despite the rapid development of CD-based optical biosensors for cardiac troponin detection, several challenges continue to limit their transition from laboratory-scale proof-of-concept systems to reliable clinical diagnostic technologies. These challenges are not restricted to analytical sensitivity alone, but involve CD synthesis and characterization, signal reproducibility, performance in complex biological matrices, multiplexed detection, clinical validation, device integration, and regulatory standardization. Addressing these issues will require coordinated advances in materials chemistry, optical sensing, device engineering, data analysis, and clinical validation.

### 6.1. Reproducibility and standardization of CDs

One of the most fundamental challenges is the lack of universally standardized procedures for the synthesis and characterization of CDs. Depending on the carbon precursor, reaction conditions, heteroatom doping, surface passivation, purification procedure, and post-synthetic modification, CDs can exhibit substantial differences in particle size, surface functional groups, electronic structure, quantum yield, excitation-dependent emission, and photostability. Consequently, two CD preparations nominally described as the same material may generate different optical responses and sensing performances. This variability is particularly important for cardiac troponin sensing because the analytical signal depends directly on the photophysical properties and surface chemistry of the CDs. Changes in functional groups can alter antibody or aptamer immobilization, target recognition, fluorescence quenching or enhancement, and nonspecific interactions with matrix components. Therefore, reporting only the synthesis procedure and nominal particle size is insufficient for establishing reproducible sensing platforms. More comprehensive characterization, including optical properties, surface composition, particle-size distribution, and batch-to-batch performance, should become an integral part of sensor development.

Future studies should therefore move toward well-defined synthesis protocols and standardized characterization criteria. Reporting quantum yield, fluorescence lifetime, excitation/emission characteristics, surface functional groups, purification procedures, and batch-to-batch variation would facilitate meaningful comparison between independent studies. Establishing such standards is particularly important if CD-based sensors are to be manufactured at larger scale and transferred between research laboratories or clinical environments. These issues represent an important barrier to the reproducibility and practical translation of CD-based.^21^

### 6.2. Complex biological matrices and nonspecific interactions

Although many CD-based optical biosensors demonstrate excellent analytical performance in buffer solutions or spiked samples, their behavior can change substantially in real biological matrices such as serum, plasma, and whole blood. Proteins, salts, lipids, metabolites, and other endogenous components may interact with CD surfaces or recognition elements, resulting in nonspecific adsorption, fluorescence quenching or enhancement, light scattering, and changes in analyte accessibility. Such matrix effects may produce differences between the apparent analytical performance obtained under controlled laboratory conditions and the actual performance in clinical samples. Antifouling strategies and appropriate surface engineering are therefore essential for improving sensor reliability. Functionalization of CDs with suitable polymers, biomolecular layers, or other protective interfaces may reduce nonspecific adsorption while preserving the accessibility of the cardiac troponin recognition element. However, surface modification can also change the optical properties and binding characteristics of CDs. Consequently, optimization should consider not only antifouling performance but also signal intensity, response time, recognition efficiency, and long-term stability. Importantly, future studies should place greater emphasis on testing in clinically relevant samples rather than relying predominantly on buffer-based experiments. Recovery, precision, interference, selectivity, and stability should be systematically evaluated using authentic patient samples and appropriate control groups. Such validation would provide a more realistic assessment of whether the high sensitivity reported for many CD-based platforms can be maintained under clinically relevant conditions.

### 6.3. Multiplexed detection of cardiac biomarkers

Another important limitation is the predominance of single-analyte sensing strategies. Although sensitive detection of cTnI or cTnT is clinically important, myocardial injury and acute cardiovascular events are complex processes that cannot always be characterized by a single biomarker. Simultaneous analysis of complementary biomarkers, such as cTnI, cTnT, BNP, and h-FABP, could provide additional information and potentially improve diagnostic discrimination. CDs are particularly attractive for multiplexed optical sensing because their emission properties can potentially be tuned through changes in size, composition, doping, and surface chemistry. Distinct emission wavelengths or fluorescence lifetimes could therefore be associated with different recognition elements. However, multiplexing introduces additional challenges, including spectral overlap, energy transfer between probes, differences in binding kinetics, and signal interference caused by biological matrices. Increasing the number of biomarkers may consequently improve the amount of diagnostic information while simultaneously increasing the complexity of signal interpretation. Future multiplexed platforms should therefore be evaluated not simply by the number of biomarkers detected, but by their ability to maintain analytical accuracy, reproducibility, and clinically meaningful classification. Ratiometric approaches, lifetime-based measurements, spectral deconvolution, and computational analysis may provide useful strategies for separating overlapping optical signals. Nevertheless, these approaches require systematic validation using well-characterized clinical samples.

### 6.4. Clinical validation and benchmarking

A major gap between current CD-based optical biosensors and established cardiac troponin assays is the limited extent of clinical validation. Many reported platforms demonstrate attractive limits of detection, broad linear ranges, rapid response times, and successful recovery in spiked samples. However, analytical sensitivity alone does not establish clinical utility. A clinically useful cardiac troponin sensor must demonstrate reliable performance across heterogeneous patient populations, different sample matrices, and clinically relevant concentration ranges. Future investigations should therefore progress beyond proof-of-concept experiments toward systematic clinical evaluation. Comparisons with established laboratory reference methods should be performed using sufficiently large numbers of clinical samples, including patients with acute myocardial infarction and appropriate non-AMI control groups. Particular attention should be given to agreement between the proposed sensor and reference assays, precision near clinically relevant decision concentrations, false-positive and false-negative rates, and robustness to common interfering substances. Large-scale and multicenter studies will ultimately be required to determine whether CD-based optical platforms provide clinically meaningful advantages over existing technologies. Such studies would also help determine whether improvements in analytical sensitivity translate into earlier diagnosis, improved risk stratification, or better clinical decision-making. Thus, future research should shift the emphasis from achieving increasingly low detection limits toward demonstrating clinical relevance and diagnostic utility.

### 6.5. Stability, manufacturing, and device integration

Long-term stability and manufacturing consistency represent additional barriers to translation. Variations in CD synthesis, recognition-element immobilization, reagent composition, and storage conditions may lead to sensor-to-sensor and batch-to-batch variability. For point-of-care applications, the sensor must also remain stable during storage, transportation, and operation under variable environmental conditions. Integration of CDs into microfluidic chips, lateral-flow formats, and portable optical readers provides a promising route toward decentralized testing. However, miniaturization can introduce new problems, including limited sample volumes, changes in fluid transport, optical background from device materials, and difficulties in maintaining uniform reagent distribution. Therefore, successful integration requires simultaneous optimization of the nanomaterial, biorecognition chemistry, optical readout, and device architecture rather than treating device fabrication as a separate final step. For practical clinical deployment, manufacturing processes should also be scalable and reproducible. The performance of laboratory-prepared sensors may not necessarily be maintained when production is increased to larger batches. Consequently, quality-control criteria for CD composition, optical properties, recognition-element loading, analytical response, and storage stability should be established before large-scale manufacturing is considered.

### 6.6. Artificial intelligence and machine-learning-assisted analysis

AI and ML provide an emerging opportunity to improve the interpretation of complex optical signals generated by CD-based biosensors. Fluorescence spectra, images, temporal signal profiles, and multiplexed optical responses may contain information that is difficult to extract using conventional single-parameter calibration. ML algorithms could assist with spectral deconvolution, pattern recognition, signal-to-noise discrimination, and quantitative prediction of cardiac troponin concentrations. Beyond signal interpretation, AI/ML may also contribute to the optimization of CD synthesis and sensor design. Relationships between precursor composition, synthesis conditions, surface chemistry, structural characteristics, and optical responses could potentially be identified from sufficiently large datasets. Such approaches may accelerate the selection of CD formulations with desired photophysical and sensing properties and could help compensate for sensor-to-sensor or batch-to-batch variability. However, AI/ML should not be considered a substitute for experimental standardization and clinical validation. Reliable models require sufficiently large, diverse, and well-annotated datasets obtained using standardized protocols. Models trained using limited laboratory datasets may perform poorly when applied to different patient populations, instruments, CD batches, or sample matrices. Interpretability, independent validation, and external testing are therefore essential before AI-assisted CD biosensors can be considered clinically reliable.^83^ Overall, the major challenge is no longer simply to demonstrate that CDs can provide sensitive optical detection of cardiac troponin. The more important question is whether these systems can provide reproducible, robust, clinically validated, and standardized measurements under real-world conditions. Addressing this transition from analytical performance to clinical utility will be essential for establishing CD-based optical biosensors as credible alternatives or complementary tools to conventional cardiac troponin testing.

## 7. Future work and perspectives

The future development of CD-based optical biosensors for cardiac troponin detection should move beyond the pursuit of lower detection limits toward reproducibility, clinical validity, multiplexed analysis, device integration, and real-world usability. The current literature demonstrates that CDs can provide strong and tunable optical responses and can be incorporated into several sensing formats; however, the evidence base remains dominated by laboratory-scale studies. The next stage of development should therefore focus on closing the gap between material innovation and clinically meaningful diagnostic performance.

### 7.1. Rational design and standardized CD synthesis

A major priority should be the development of reproducible CD synthesis strategies that allow predictable control over particle size, surface chemistry, electronic structure, and photoluminescence. Rather than optimizing each CD formulation empirically, future studies should establish clearer relationships between synthesis parameters, physicochemical characteristics, and sensing responses. Controlled heteroatom doping, surface passivation, and post-synthetic functionalization may provide routes for tuning emission intensity, wavelength, lifetime, and recognition-element immobilization. Standardized reporting will also be essential. Future studies should provide sufficient information on precursor composition, reaction conditions, purification, structural characterization, optical properties, and batch-to-batch variation. Such reporting would make it easier to reproduce promising sensing systems and compare results obtained by different research groups. Ultimately, CD synthesis should progress toward well-defined and scalable processes suitable for manufacturing rather than relying exclusively on laboratory-specific preparation procedures.

### 7.2. From analytical sensitivity to clinical utility

An important shift in future research should be from reporting increasingly low limits of detection toward demonstrating clinically relevant diagnostic performance. A sensor may achieve an extremely low analytical detection limit but still have limited clinical value if it exhibits poor reproducibility, matrix interference, inadequate selectivity, or insufficient agreement with established clinical assays. Future studies should therefore evaluate CD-based sensors at clinically relevant cardiac troponin concentrations and compare their results directly with validated reference methods. Studies should include appropriate patient groups and should report parameters such as precision, accuracy, recovery, interference, agreement, diagnostic sensitivity, and specificity. Large clinical cohorts and multicenter validation will be particularly important for determining whether the advantages observed in laboratory studies are maintained across different populations, sample types, and testing environments.

This transition is essential because the ultimate value of a cardiac troponin biosensor should be determined by its ability to support clinical decision-making rather than by analytical performance alone. Accordingly, future investigations should ask whether a CD-based optical platform can provide sufficiently rapid, reliable, and clinically interpretable information to improve early assessment of myocardial injury.

### 7.3. Multiplexed cardiac biomarker detection

Future CD-based platforms should increasingly move toward multiplexed detection. Simultaneous measurement of cTnI or cTnT together with complementary biomarkers such as BNP and h-FABP could provide a more comprehensive biochemical profile of cardiovascular injury. CD-based optical probes are potentially suitable for such applications because their optical properties can be engineered to generate distinguishable signals. However, successful multiplexing will require more than simply combining several probes in one assay. Spectral overlap, differences in biomarker concentration, cross-reactivity, competition for binding sites, and matrix-dependent signal changes must be carefully controlled. Ratiometric fluorescence, fluorescence lifetime analysis, spectrally resolved detection, and computational signal separation may help overcome these limitations. The most valuable future studies should therefore evaluate whether multiplexing actually improves diagnostic classification compared with single-biomarker measurement. In other words, the emphasis should shift from demonstrating technical multiplexing to demonstrating clinically meaningful information gained from multiplexed analysis.

### 7.4. Microfluidic, lateral-flow, and smartphone-assisted platforms

The integration of CD-based optical sensors with miniaturized platforms represents one of the most promising pathways toward point-of-care testing. Microfluidic systems can reduce sample and reagent consumption while enabling controlled sample handling and sequential assay steps. Lateral-flow configurations may provide simpler operation and rapid visual or optical readout, whereas smartphone-assisted systems could offer accessible image acquisition and quantitative analysis. Nevertheless, device integration should be designed together with the sensing chemistry. Optical background, illumination variability, camera characteristics, environmental conditions, sample volume, and fluid transport can all affect the measured signal. Future studies should therefore evaluate the complete sensing system rather than reporting the performance of the CD probe independently from the device. For smartphone-assisted systems in particular, calibration across different smartphone cameras and lighting conditions will be important. Standardized optical interfaces and reference standards could help reduce device-dependent variability. The ultimate goal should be the development of compact systems that can perform sample processing, biomarker recognition, signal generation, and quantitative interpretation with minimal user intervention.

### 7.5. AI/ML-assisted optical biosensing

AI and ML are expected to become increasingly relevant as CD-based sensors evolve toward multiplexed and image-based detection. Machine-learning models could assist in extracting quantitative information from fluorescence spectra, colorimetric images, chemiluminescence signals, or combinations of multiple biomarkers. Such approaches may be particularly useful when signals overlap or when matrix effects complicate conventional calibration. However, AI integration should be approached cautiously. A model developed using a small dataset generated from a single CD batch or experimental platform may not generalize to different laboratories or patient populations. Future work should therefore prioritize large and diverse datasets, standardized data acquisition, external validation, and interpretable models. Combining optical biosensors with AI should ultimately improve robustness and clinical decision support rather than simply increase computational complexity.^83^

### 7.6. CD–MOF hybrid systems and advanced nanostructures

The combination of CDs with MOFs and other functional nanomaterials provide another promising direction. MOFs can offer high surface area, tunable porosity, and abundant sites for molecular interaction, while CDs can contribute favorable optical properties. CD–MOF hybrid structures may therefore provide opportunities for improved analyte enrichment, signal amplification, and optical transduction. Related advances in MOF-based and CD–MOF sensing systems support the potential of these hybrid architectures performance.^84^ Nevertheless, future research should establish whether the additional structural complexity of CD–MOF hybrids produce a meaningful analytical or clinical advantage compared with simpler CD-based systems. Hybrid materials should therefore be evaluated not only according to sensitivity but also in terms of synthesis reproducibility, stability, biocompatibility, scalability, cost, and ease of integration into portable devices. Demonstrating a clear benefit over existing CD platforms will be necessary to justify their further development.

### 7.7. Manufacturing, standardization, and regulatory translation

Clinical translation will ultimately depend on whether CD-based biosensors can be manufactured consistently and evaluated according to appropriate quality and regulatory requirements. Laboratory-scale synthesis may be sufficient for proof-of-concept experiments but is unlikely to meet the reproducibility requirements of clinical diagnostics without further optimization. Future research should therefore incorporate manufacturing considerations at an early stage. This includes scalable CD synthesis, standardized surface functionalization, quality-control procedures, shelf-life assessment, device-to-device reproducibility, and defined acceptance criteria for analytical performance. Regulatory requirements should also be considered during platform development rather than only after the sensor has been fully optimized. Interdisciplinary collaboration between materials scientists, analytical chemists, engineers, data scientists, and clinicians will be essential for addressing these challenges. Such collaboration can ensure that material innovations are evaluated against clinically relevant requirements and that device development is guided by actual diagnostic needs.

### 7.8. Overall perspective

Taken together, the future of CD-based optical biosensors for cardiac troponin detection should be defined by a transition from proof-of-concept sensing toward clinically validated diagnostic systems. The most promising platforms are likely to combine reproducibly synthesized CDs, selective biorecognition elements, robust optical transduction, multiplexed biomarker analysis, miniaturized sample handling, portable readout, and validated computational interpretation.

Importantly, progress should not be measured solely by lower detection limits or increasingly complex nanostructures. Instead, future research should prioritize reproducibility, clinically relevant accuracy, robustness in real biological matrices, scalability, usability, and agreement with established clinical methods. If these requirements can be systematically addressed, CD-based optical biosensors could progress from promising laboratory technologies toward practical point-of-care tools for rapid cardiac biomarker assessment.^85^

## 8. Conclusions

In this review, we have outlined the recent advances in CDs-based optical biosensors for cardiac troponin detection, highlighting their growing potential as sensitive, rapid, and versatile diagnostic platforms. Fluorescence-, colorimetric-, and chemiluminescence-based sensing strategies have demonstrated significant improvements in analytical performance compared with conventional approaches, offering advantages such as ultra-low detection limits, shorter assay times, and suitability for portable and point-of-care applications. These attributes position CDs-based biosensors as promising candidates for the early diagnosis and monitoring of cardiovascular diseases. Despite these encouraging developments, several challenges continue to hinder their clinical translation. Variations in CD synthesis methods affect reproducibility and comparability across studies, while limited validation in complex biological samples raises concerns regarding their practical applicability in clinical settings. Furthermore, issues related to long-term stability, scalable manufacturing, regulatory approval, and standardization remain important obstacles to commercialization. The absence of fully integrated and multiplexed diagnostic platforms also limits the broader clinical utility of current systems. Looking ahead, continued progress will require the establishment of standardized synthesis and characterization protocols, comprehensive validation using clinically relevant samples, and the integration of CDs-based biosensors with portable and digital healthcare technologies. Advances in multiplexed sensing, automated analysis, and data-driven diagnostic approaches are expected to further enhance sensitivity, accuracy, and clinical relevance. Overall, CDs-based optical biosensors represent a highly promising direction for next-generation cardiac diagnostics; however, their successful transition from laboratory research to routine clinical practice will depend on coordinated efforts in materials design, device engineering, and rigorous clinical validation.

## Author contributions

Shilan A. Fatah: writing – original draft, investigation, formal analysis, data curation, conceptualization. Aryan A. Fatah: visualization, validation, software. Elyan A. Abdulla: data collection, methodology, writing – original draft. Sanaa K. Ali: formal analysis, methodology, writing – original draft. Rzgar Sirwan Raza: software, visualization, investigation. Sameera Sh. M. Ameen: conceptualization, investigation, Khalid M. Omer: writing – review & editing, supervision, conceptualization.

## Conflicts of interest

The authors declare that they have no known competing financial interests or personal relationships that could have appeared to influence the work reported in this paper.

## Data Availability

Data will be made available on request.

## References

[cit1] Arnett D. K., Blumenthal R. S., Albert M. A., Buroker A. B., Goldberger Z. D., Hahn E. J., Himmelfarb C. D., Khera A., Lloyd-Jones D., McEvoy J. W., others (2019). J. Am. Coll. Cardiol..

[cit2] Bajaj A., Sethi A., Rathor P., Suppogu N., Sethi A. (2015). J. Invest. Med..

[cit3] Vander Borght M., Wyns C. (2018). Clin. Biochem..

[cit4] Xu Y.-J., Zheng L., Hu Y.-W., Wang Q. (2018). Clin. Chim. Acta.

[cit5] Kim Y. J., Yun J., Il Kim S., Hong H., Park J.-H., Pyun J.-C., Ryu W. (2018). Biosens. Bioelectron..

[cit6] Zou Y., Li M., Xing Y., Duan T., Zhou X., Yu F. (2019). ACS Sens..

[cit7] Du X., Su X., Zhang W., Yi S., Zhang G., Jiang S., Li H., Li S., Xia F. (2021). Anal. Chem..

[cit8] Fathil M. F. M., Arshad M. K. M., Ruslinda A. R., Nuzaihan M., Gopinath S. C. B., Adzhri R., Hashim U. (2016). Anal. Chim. Acta.

[cit9] Sh S., Ameen M., Fatah S. A., Algethami F. K., Omer K. M. (2026). J. Mater. Sci..

[cit10] Bedair A., Hamed M., Mohammed Ameen S. Sh., Omer K. M., Mansour F. R. (2024). Microchem. J..

[cit11] Li X., Guo T., Li M., Yang C., Zhou H., Liu Y., Iqbal S., Huang Z., Darwish M. A., Salem M. M., Zhou T., Zhou D. (2026). Carbon.

[cit12] Qin H., Dai H., Wu M., Zhang Z., Wang Y. (2026). Nanoscale.

[cit13] Lian M., Wang H., Chen L., Chen D., Yang Q., Qiao X. (2025). Sens. Actuators, B.

[cit14] Du D., Shu J., Guo M., Haghighatbin M. A., Yang D., Bian Z., Cui H. (2020). Anal. Chem..

[cit15] Zhao Q., Song W., Zhao B., Yang B. (2020). Mater. Chem. Front..

[cit16] Li L., Dong T. (2018). J. Mater. Chem. C.

[cit17] Moon H., Park B., Hong D., Park K.-S., Lee S., Kim S. (2021). Sens. Actuators, B.

[cit18] Sokolowska P., Zukowski K., Janikiewicz J., Jastrzebska E., Dobrzyn A., Brzozka Z. (2021). Biosens. Bioelectron..

[cit19] Huang X., Ren J. (2012). TrAC, Trends Anal. Chem..

[cit20] Yang S.-M., Lv S., Zhang W., Cui Y. (2022). Sensors.

[cit21] Mohammed Ameen S. Sh., Bedair A., Hamed M., Mansour F. R., Omer K. M. (2024). Microchem. J..

[cit22] Sh S., Ameen M., Algethami F. K., Omer K. M. (2026). J. Inorg. Organomet. Polym. Mater..

[cit23] Qader I. B., Ameen S. Sh. M., Qader H. A., Omer K. M. (2026). ChemistrySelect.

[cit24] Qureshi Z. A., Dabash H., Ponnamma D., Abbas M. K. G. (2024). Heliyon.

[cit25] Christenson E., Christenson R. H. (2013). Ann. Lab. Med..

[cit26] Lai J. S. Y., Yeong N., Tan X. Y., Chen M., Ng I. K. S., Teo D. B., Ong L., Lim S. L. (2026). Am. Med. J..

[cit27] V Gomes A., Potter J. D., Szczesna-Cordary D. (2002). IUBMB Life.

[cit28] White S. P., Cohen C., Phillips Jr G. N. (1987). Nature.

[cit29] Fathil M. F. M., Arshad M. K. M., Gopinath S. C. B., Hashim U., Adzhri R., Ayub R. M., Ruslinda A. R., Nuzaihan M., Azman A. H., Zaki M., others (2015). Biosens. Bioelectron..

[cit30] Katrukha I. A., Katrukha A. G. (2021). Clin. Chem..

[cit31] Tiwari R. P., Jain A., Khan Z., Kohli V., Bharmal R. N., Kartikeyan S., Bisen P. S. (2012). Mol. Diagn. Ther..

[cit32] Hayat H., Awan F. R., Schirhagl R., Saeed M. H., Khan W. S., Bajwa S. Z. (2026). J. Mater. Res..

[cit33] Bai H., He L., Liu J. H., Liu Z. J., Ren J. T., Wang E. K. (2023). Chin. J. Anal. Chem..

[cit34] Li J., Zhang S., Zhang L., Zhang Y., Zhang H., Zhang C., Xuan X., Wang M., Zhang J., Yuan Y. (2021). Front. Chem..

[cit35] Gupta A., Sharma S. K., Pachauri V., Ingebrandt S., Singh S., Sharma A. L., Deep A. (2021). RSC Adv..

[cit36] Xia L., Yang J., Su R., Zhou W., Zhang Y., Zhong Y., Huang S., Chen Y., Li G. (2019). Anal. Chem..

[cit37] Yadav P., Divya N., Srivastava V., Saran A. D. (2026). Chem. Pap..

[cit38] Paveethra S., Manisekaran H., Sasidharan S. (2024). Asian Pac. J. Cancer Prev..

[cit39] Gao Y.-Y., He J., Li X.-H., Li J.-H., Wu H., Wen T., Li J., Hao G.-F., Yoon J. (2024). Chem. Soc. Rev..

[cit40] Kang Z., Lee S.-T. (2019). Nanoscale.

[cit41] Yu J., Yong X., Tang Z., Yang B., Lu S. (2021). J. Phys. Chem. Lett..

[cit42] Sharma A., Das J. (2019). J. Nanobiotechnol..

[cit43] Zhou Y., Zhang W., Leblanc R. M. (2022). J. Phys. Chem. B.

[cit44] Li Z., Wang L., Li Y., Feng Y., Feng W. (2019). Mater. Chem. Front..

[cit45] Liu M. L., Bin Chen B., Li C. M., Huang C. Z. (2019). Green Chem..

[cit46] Xu Q., Kuang T., Liu Y., Cai L., Peng X., Sreenivasan Sreeprasad T., Zhao P., Yu Z., Li N. (2016). J. Mater. Chem. B.

[cit47] Ozyurt D., Al Kobaisi M., Hocking R. K., Fox B. (2023). Carbon Trends.

[cit48] Wu Z. L., Liu Z. X., Yuan Y. H. (2017). J. Mater. Chem. B.

[cit49] Chahal S., Macairan J.-R., Yousefi N., Tufenkji N., Naccache R. (2021). RSC Adv..

[cit50] Li H., Kang Z., Liu Y., Lee S.-T. (2012). J. Mater. Chem..

[cit51] Zhu S., Song Y., Zhao X., Shao J., Zhang J., Yang B. (2015). Nano Res..

[cit52] Han Z., He L., Pan S., Liu H., Hu X. (2020). Luminescence.

[cit53] Zhou M., Zhou Z., Gong A., Zhang Y., Li Q. (2015). Talanta.

[cit54] Baker S. N., Baker G. A. (2010). Angew. Chem., Int. Ed..

[cit55] Lim S. Y., Shen W., Gao Z. (2015). Chem. Soc. Rev..

[cit56] Chen Q., Wu W., Wang K., Han Z., Yang C. (2023). J. Thorac. Dis..

[cit57] Han G.-R., Kim M.-G. (2020). Sensors.

[cit58] Gogoi S., Khan R. (2018). Phys. Chem. Chem. Phys..

[cit59] Ozdemir A., Algül Y., Tirgil N. Y. (2025). J. Fluoresc..

[cit60] Liu M., Jiang R., Zheng M., Li M., Yu Q., Zhu H., Guo H., Sun H. (2022). Talanta.

[cit61] Lakshmanakumar M., Nesakumar N., Sethuraman S., Rajan K. S., Krishnan U. M., Rayappan J. B. B. (2019). Sci. Rep..

[cit62] Kim S. E., Yoon J. C., Muthurasu A., Kim H. Y. (2024). Sens. Actuators, B.

[cit63] Kim S. E., Yoon J. C., Muthurasu A., Kim H. Y. (2024). Langmuir.

[cit64] Varghese S., Madanan A. S., Abraham M. K., Shkhair A. I., Indongo G., Rajeevan G., Vijila N. S., George S. (2023). Microchem. J..

[cit65] Memon R., Shaheen I., Qureshi A., Niazi J. H. (2024). J. Alloys Compd..

[cit66] Chen J. N., Hasabnis G. K., Akin E., Gao G., Usha S. P., Süssmuth R., Altintas Z. (2024). Sens. Actuators, B.

[cit67] Radha R., Shahzadi S. K., Al-Sayah M. H. (2021). Molecules.

[cit68] Heidt B., Siqueira W. F., Eersels K., Diliën H., van Grinsven B., Fujiwara R. T., Cleij T. J. (2020). Biosensors.

[cit69] Vealan K., Joseph N., Alimat S., Karumbati A. S., Thilakavathy K. (2023). Asian Biomed..

[cit70] Huang Y., Li M., Sun J., Suo Z., Lu T., Wei M., Jin H. (2025). Microchem. J..

[cit71] He Y., Qu X., Lan T., Yu M., Duan X., Chen R., Wu J., Wang Z., Kong D., Shen Z., others (2025). Sens. Biosens. Res..

[cit72] Ulloa-Gomez A. M., Agredo A., Lucas A., Somvanshi S. B., Stanciu L. (2023). Biosens. Bioelectron..

[cit73] Saha K., Agasti S. S., Kim C., Li X., Rotello V. M. (2012). Chem. Rev..

[cit74] Han Y. D., Chun H. J., Yoon H. C. (2014). Biosens. Bioelectron..

[cit75] Miao L., Jiao L., Tang Q., Li H., Zhang L., Wei Q. (2019). Sens. Actuators, B.

[cit76] Abbasi R., Hu X., Zhang A., Dummer I., Wachsmann-Hogiu S. (2024). Bioengineering.

[cit77] Zubair M. and Sharma S., Analytical and Clinical Aspects of Troponin Testing, in StatPearls, StatPearls Publishing, Treasure Island, FL, 2026 29939582

[cit78] Wang Y., Fill C., Nugen S. R. (2012). Biosensors.

[cit79] Ashraf A. A., Rai S., Alva S., Alva P. D., Naresh S. (2025). Diagn. Microbiol. Infect. Dis..

[cit80] Yang R., Li F., Zhang W., Shen W., Yang D., Bian Z., Cui H. (2019). Anal. Chem..

[cit81] Kricka L. J. (1991). Clin. Chem..

[cit82] Miao W. (2008). Chem. Rev..

[cit83] Raza R. S., Fatah S. A., Ameen S. S. M., Omer K. M. (2026). Talanta.

[cit84] Fatah S. A., Ameen S. Sh. M., Hassan P. B., Omer K. M. (2026). Microchim. Acta.

[cit85] Mansour F. R., Omer K. M., Ameen S. Sh. M., Locatelli M., Ali I., Bedair A. (2026). Analytica.

